# When daily activities become digitally curated: a perspective through the model of human occupation

**DOI:** 10.3389/fpsyg.2026.1940839

**Published:** 2026-09-11

**Authors:** Nevita Saha, Latika Pandey, Shefali Parmar

**Affiliations:** Department of Occupational Therapy, Yenepoya School of Allied Health Sciences, Yenepoya (Deemed to be University), University Road, Mangalore, Karnataka, India

**Keywords:** occupational disruption, occupational imbalance, occupational science, smartphone addiction, social media usage

## Abstract

Dopamine-scrolling characterized by prolonged engagement with algorithmically curated social media content driven by intermittent rewards and personalized recommendations has emerged as a common pattern of digital behavior with potential implications for everyday occupations. Although dopamine-scrolling has been discussed in relation to psychological constructs, its influence on occupational participation has received limited attention. This perspective article draws on broader literature on Social Media Use (SMU) and applies the Model of Human Occupation to conceptualize dopamine-scrolling as an emerging occupational phenomenon. It proposes that prolonged engagement with algorithmically curated digital environments may reshape occupational participation by altering volitional processes and promoting passive occupational participation, shifting occupational identity towards performative occupational engagement, and contributing to occupational imbalance through the disruption or displacement of meaningful occupations, with potential consequences for health and wellbeing. This perspective reframes dopamine-scrolling as a phenomenon capable of reshaping occupational participation, occupational engagement, occupational identity and occupational balance. It also highlights the societal implications and recommendations for future research, occupational therapy practice, interdisciplinary collaborations and digital literacy initiatives aimed at promoting more intentional and meaningful patterns of digital participation. The concepts presented are intended as interpretive propositions requiring empirical validation across populations and contexts.

## 1 Introduction

The World Federation of Occupational Therapists defines occupations as not merely employment but “the everyday activities that people do as individuals, in families and with communities to occupy time and bring meaning and purpose to life. These include things people need to, want to and are expected to do” (WFOT, n.d.). They are linked to an individual’s identity, self-competence, belongingness, and life purpose deriving their significance from subjective experiences of doing, being, belonging, and becoming (Hitch et al., 2018). Accordingly, “meaning” stems from perceived importance and occupational engagement within a personalized context. Individuals experience a sense of agency when they are able to choose their occupations in order to align them with their identity, roles, and life goals (Granholm Valmari et al., 2024). In contrast, individuals who have addictions or impulse-control disorders may become pre-occupied in certain activities which occupy a major part of their time and attention diminishing the individual’s capacity for making meaningful choices (Pellegrino et al., 2022). Specific narratives, glorified identities, social desirability, and branding may be constructed by social media which influences how individuals understand themselves, their activities, and consequently their communities (Lathan, 2026). In this way, social media actively shapes the meaning, value, visibility, and desirability of occupations.

Algorithms, a dominant feature of digital platforms, curate content, customize feeds, and influence daily interactions and experiences (Baidya et al., 2023; Ionescu and Licu, 2023; Chung and Wihbey, 2024). Social media platforms employ artificial intelligence-driven systems that continuously analyze user behavior and interaction patterns leading to personalized content and optimized engagement. Features such as auto-play, infinite scrolling and personalized recommendations minimize opportunities for disengagement (De et al., 2025). These systems shape how individuals access information, communicate and participate in digital activities through gig-work apps, healthcare tools, and adaptive learning systems, in addition to streamlining tasks such as shopping, navigation, and communication (Baidya et al., 2023). Social media use (SMU), which can refer to the time spent using social media platforms (Bekalu et al., 2023), frequently begins with a clear aim. However, platforms deliberately encourage users to keep scrolling by rewarding them with erratic bursts of novelty and enjoyment, a phenomenon termed as “dopamine-scrolling” (Sharpe and Spooner, 2025). Consequently, excessive SMU may impair emotional regulation, sleep quality, physical health, and interpersonal relationships. Health consequences include elevated stress, anxiety, and depression linked to impaired sleep, decreased physical activity, or mental health conditions related to addiction, impaired attention, reduced working memory, poorer executive functioning, and impaired decision-making (Naik et al., 2025; Lahti et al., 2024; Nawaz et al., 2024).

The Model of Human Occupation (MOHO), an evidence-based and client-centred model, is used to propose how SMU potentially alters occupational functioning. Occupational therapists apply MOHO to understand “what motivates a person to action” (volition), what forms their habits (habituation), what their subjective lived experiences are (performance capacity) in relation to their environmental contexts (Taylor and Kielhofner, 2017; Kielhofner and Burke, 1980; Kielhofner and Forsyth, 1997; Lee et al., 2012). It comprises of volitional processes which organize and direct occupational choices and give everyday occupations meaning. Due to social media’s engaging design, digital usage may become increasingly driven by reinforcement mechanisms rather than guided by personal values or purposeful intent. Environmental and contextual factors may also restrict autonomy and reduce opportunities for occupational choice, resulting in situations where individuals remain active yet their participation is shaped by external rules and constraints (Ali et al., 2025; Kirschner et al., 2023). When such environmental influences begin to interfere with individual’s ability to engage in occupations aligned with their values and intentions, they may contribute to occupational disruption (Nizzero et al., 2017).

**Figure 1:**
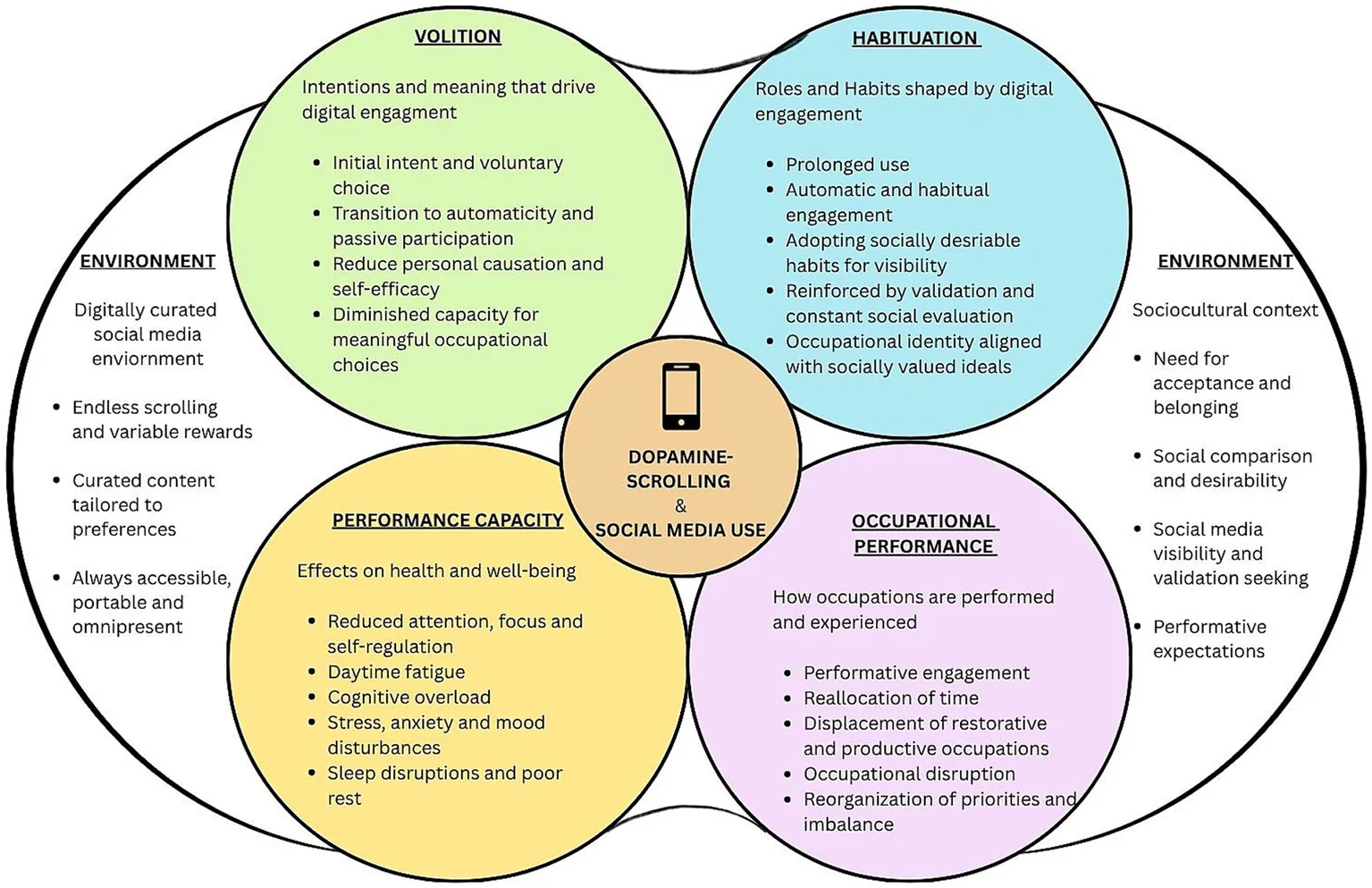
Proposed conceptual model of dopamine-scrolling and SMU through the lens of the MOHO. The model illustrates how algorithms interact with the MOHO subsystems of volition, habituation, performance capacity, and occupational performance. The digital and sociocultural environments are omnipresent and engulf all MOHO subsystems. The three interrelated processes described in this perspective are presented within the MOHO subsystems.

As of 2023, globally, the number of younger social media users (mean age 35.79) surpasses non-users (mean age 45.47) with more women users (52%) (Shahghasemi et al., 2025). SMU is an increasingly ubiquitous component of daily life and social media is used for daily activities like communication, social participation, or to fill otherwise unoccupied moments during commuting and waiting in lines (Trifiro and Gerson, 2019). Accordingly, drawing on broader literature on SMU given the limited specific evidence, this perspective proposes that dopamine-scrolling should be understood as an emerging occupational phenomenon through which algorithmically reinforced digital participation progressively reshapes occupational participation. We propose that this occurs through three interrelated processes: (1) alteration of volitional processes and passive participation, (2) reconstruction of occupations, occupational identity leading to performative occupational engagement (3) occupational imbalance and consequences of displaced meaningful occupations (Figure 1).

## 2 Alteration of volitional processes and passive participation

### 2.1 From volition to automaticity

According to MOHO, occupation is fundamentally driven by volition, which reflects an individual’s motivation for occupation and includes personal causation, values and interests (Taylor and Kielhofner, 2017). These volitional subsystems guide occupational choice through an ongoing process of anticipating, choosing, experiencing and interpreting occupations, making participating an active reflective process through which individuals develop occupational competence, identity and derive meaning from everyday activities (Taylor and Kielhofner, 2017). Dopamine-scrolling could challenge these volitional processes.

The algorithm-driven digital landscape does not require conscious decisions to continue usage. Rather SMU becomes passive as the platform’s features allow users to transition seamlessly from one piece of content to another, often without temporal awareness (De et al., 2025). Each scroll presents the possibility of encountering content perceived as humorous, emotionally salient, socially rewarding or personally relevant. The associated unpredictability encourages continued SMU not because of the content’s inherent meaning but because each interaction carries expectation of potential reward (Sharpe and Spooner, 2025). Individuals may consciously engage with the digital platform for a specific purpose; however, the algorithmically generated personalized content redirects attention, making SMU progressively less intentional. Over time, such occupational participation may influence the volitional system itself. Individuals who repeatedly perceive themselves as unable to disengage from prolonged scrolling may experience diminished personal causation reflected in reduced self-efficacy to regulate their occupational participation. At the same time, a discrepancy might exist between meaningful occupations and SMU patterns which weakens the congruence between value and behavior. Similarly, dopamine-scrolling may replace intrinsically motivated and interest-based activities, shifting selection of meaningful activities to those that are digitally present.

### 2.2 Habitual passive digital participation

From an occupational science perspective, dopamine-scrolling may represent a passive form of participation in which a person’s choice to use social media becomes limited. This is in contrast to intentionally initiating, sustaining, and experiencing occupations meaningfully. It is also different from “passive social media use” in which individuals on social media platforms browse without reacting to or engaging with the online content (Beyens et al., 2024). Once SMU becomes passive, it is no longer actively directed but automatically performed and shaped by the digital environment (De et al., 2025). This continued scrolling extends beyond the individual’s original intention and may alter how time is allocated across daily occupations and routines thus influencing the habituation subsystem within MOHO.

Prolonged passive SMU may reduce opportunities for deliberate occupational decision making. Time that may have been allocated to productive, recreational, social or self-care occupations becomes occupied by ongoing digital consumption. Thus, occupational choices are not necessarily eliminated but are displaced, as algorithmically reinforced participation competes with occupations that are more closely aligned with an individual’s values and holds meaning.

## 3 Reconstruction of occupations, occupational identity leading to performative occupational engagement

### 3.1 Occupational identity and desirability

The MOHO describes occupational identity as a composite sense of who one is and wishes to become as an occupational being developing through occupational participation as individuals engage in roles and habits, and accumulate experiences that shape their sense of competence, values, and self-concept (Kielhofner, 2008). With the rapid integration of SMU into everyday life, “meaning” transforms potential digital representation. Social media platforms are continuously observed, evaluated, documented, and compared, altering how individuals construct their occupational identities.

Instagram has been conceptualized as a continuous “identity work” where individuals repeatedly negotiate, reconstruct and question their sense of self through interactions with algorithms and social expectations. Identity is therefore viewed as an ongoing process of becoming which extends beyond active platform use as emotional responses and self-evaluations continue to influence individuals after they disengage from scrolling (Soini and Lafaire, 2025). This influence may extend into offline occupational choices, as digitally constructed identities may shape perceptions of desirable, meaningful or worthwhile activities. Therefore, daily activities may no longer be meaningfully chosen but done because they are perceived as socially desirable or worthy of sharing online. The anticipated documentation of an occupation becomes integrated into the occupation itself. This conceptual shift aligns with findings demonstrating that problematic SMU is driven less by entertainment and more by motives such as self-status, social compensation and escape. Yet, individuals with offline social difficulties, escaping negative emotional experiences, are more likely to exhibit symptoms of problematic SMU (Thorell et al., 2024). Therefore, digital participation may often be motivated by attempts to construct, maintain or validate occupational identity rather than recreation alone.

### 3.2 From authentic to performative occupational engagement

Social media platforms encourage individuals to present aspects of their everyday lives into an audience. As occupations become visible online for social evaluation, they may be experienced for how they are perceived by others. For example, followers of lifestyle influencers negotiate meanings of success, self-worth, and identity through interactions with materialistic influencer content. Hence, individuals interpret influencer-promoted products and lifestyle functions as social symbols rather than merely commercial advertisements. This shapes how followers perceive themselves and their consumption choices through symbolic presentations of desirable lifestyles and internalize associated meanings through admiration, aspiration, comparison and envy. Consumption decisions emerge not simply from advertising exposure but from attempts to align one’s identity with socially valued ideals (Azhar et al., 2025). However, this raises broader questions regarding digital participation beyond consumption. Continuous exposure to curated lifestyles, social norms, and digitally reinforced ideals may shape how users perceive success, relationships, productivity, leisure and everyday participation. Individuals may begin selecting or modifying occupations not solely because they are personally meaningful, but because they align with socially valued narratives or are anticipated to receive visibility online.

This may represent a transition from authentic towards performative occupational engagement. Where the former is primarily organised around personal meaning, enjoyment, necessity or participation, the latter may be organised around visibility, audience engagement, and anticipated social evaluation. Participants may therefore become guided by questions such as “*Will doing this be worth posting?*,” *“Will this strengthen how others perceive me*?,” or “*Will this reflect the person I want to appear to be?.”* This does not imply that social media inevitably diminishes occupational meaning. Digital platforms may facilitate creativity, social connection, advocacy, learning and community participation. Nevertheless, the continuous opportunities for performative engagement may introduce an influence on occupational identity.

## 4 Occupational imbalance: consequences of displaced meaningful occupations

Occupational balance refers to a healthy mix of various daily activities in which individuals engage and which leads to wellbeing (Hasani Helm et al., 2025; Wilcock and Hocking, 2024). When engagement is inappropriate, stress or boredom leads to occupational imbalance (Park et al., 2021).

### 4.1 Displacement of meaningful occupations

Time and energy are limited for daily activities. As dopamine-scrolling increases, these resources are gradually diverted from self-care, productivity, leisure, and social interactions to passive SMU. Scrolling during transitional periods, such as before sleep, between chores, or during a commute, can take over spaces that were previously used for restorative or productive occupations like reading, reflection, or preparation for the next day (Nasirudeen et al., 2017). This displacement may be particularly evident when scrolling extends into periods traditionally reserved for rest, contributing to delayed sleep onset, reduced sleep duration and daytime fatigue. This suggests that continuous SMU often replaces time allocated to rest and recuperate (Yu et al., 2024). From an occupational science perspective, the occupations most vulnerable to this displacement are often those that contribute to identity, belonging, and life purpose, such as participating in roles and occupations of parenting, volunteering, work, creative pursuits or social relationships, as they compete with the immediate reinforcement provided by dopamine-scrolling (Hocking, 2024). Over time, passive digital participation gradually rearranges the daily occupational pattern so that unstructured and transitional moments are increasingly filled by scrolling rather than by occupations that support health (Sharpe and Spooner, 2025). Displacement does not represent loss of time, rather it may represent a gradual reorganisation of occupational priorities.

### 4.2 Occupational disruption

Occupational disruption refers to a “state of impaired performance which usually resolves itself given supportive conditions” (Whiteford, 2000). It represents a restriction or alteration in occupational performance due to unwanted or varying contextual factors (Nizzero et al., 2017; Muriithi and Bay, 2024). Persistent occupational imbalance can render individuals unable to align their occupational choices with their values leading to occupational disruption (Nizzero et al., 2017). Hence, dopamine-scrolling and SMU, may be conceptualized as a contextual influence that restricts or alters meaningful occupations. On the contrary, smartphones and SMU pervade daily occupations such as travel, work, communication and social participation allowing individuals to adapt activities in response to their changing circumstances (Christin et al., 2014). In these cases, they function as occupational enhancers supporting individuals in their occupations thus creating a fine line between enhancing and restricting occupational performance.

Nowadays smartphone often occurs alongside other work-related digital devices (Heitmayer, 2022). It repeatedly interrupts work, leisure, and household tasks acting as a “cognitive attractor” that diverts attention from the primary activity. A study on smartphone multi-tasking in university students showed that the occupation of studying suffered repeated interruptions (Zhou and Deng, 2024). Consequently, this alters the quality of participation reducing engagement in interpersonal interactions and displacing occupations that are more aligned with an individual’s interests and values. Such disruptions may also manifest through risky behaviors, for example, unsafe behaviors like smartphone use while driving (Oviedo-Trespalacios et al., 2019).

Smartphone use may negatively impact others during co-occupations. Parents’ smartphone-use during occupations with children, like caregiving, meal preparation, play, and bedtime, was associated with slower language development (Sundqvist et al., 2021). It impairs the quality of parent–child interactions by disrupting conversational turn-taking and responsiveness. In co-occupations, couples report that excessive phone use diverts attention from interpersonal engagement, contributing to ‘phubbing’ (Frackowiak et al., 2025).

## 5 Recommendations and future implications

### 5.1 Societal implication

If the proposed conceptual pathways are established in future qualitative or empirical studies, then algorithmically curated SMU may extend beyond individual just screen time or mental health outcomes. Broader patterns of social participation and occupational functioning are affected due to continuous exposure to algorithmic SMU which may alter the salience and persistence of responses to socially significant events. This could shape how individuals demonstrate support, affiliation, and social responsibility. In this context, participation itself may become increasingly performative, with visible online responses functioning as markers of social belonging or moral alignment.

Within occupations, the increasing visibility and social evaluation of daily activities may shift participation toward activities perceived as desirable, shareable, or identity-enhancing. This may have implications for occupational identity, belonging, and occupational balance when participation becomes increasingly guided by external validation rather than personal meaning. These possibilities may be particularly relevant during childhood and adolescence, when occupational identity and routines are developing, and among individuals whose opportunities for social participation are already shaped by disability or other contextual factors.

These implications also suggest that digital wellbeing interventions should extend beyond limiting duration of use. Health features in smartphones such as screen-time reminders may interrupt excessive SMU, but may not address the deeper processes through which algorithmically curated environments influence occupations. A broader occupational approach may therefore consider not only how long individuals engage with digital media, but what that engagement is replacing, what meaning it provides, and how it shapes participation in everyday life.

If these proposed mechanisms operate cumulatively across individuals, they may contribute to population-level shifts in occupational patterns, including increasing prioritization of digitally mediated occupations, reduced participation in restorative and productive occupations, and changing norms around how everyday activities are valued and performed.

At the individual level, however, individuals may consequently turn to the same digital environment not only for entertainment or information, but also to regulate distress, seek explanations for their experiences, maintain connection, and determine what requires attention. Over time, this may contribute to a growing dependence on digitally mediated cues for understanding oneself and navigating everyday life. Individuals with mental health conditions and acquired disabilities may face occupational imbalances and disruptions due to their conditions. Excessive SMU may exacerbate occupational dysfunctions by occupying time which otherwise could be allotted to meaningful activities, altering occupational choices due to social desirability and visibility. Technology developers need to consider such vulnerable population while implementing wellbeing initiatives.

The literature in this perspective mostly encompasses SMU patterns and health consequences of healthy young adults, adolescents, and children, whereas other age groups and populations lack representation. Further study may explore the experiences and effects of SMU and dopamine-scrolling on various at-risk population such as older adults or individuals with acquired or congenital conditions or disabilities.

### 5.2 Implications for future research

Future empirical and qualitative research should examine occupational participation, engagement, balance and disruption associated with SMUs and dopamine-scrolling. Interventions may benefit from facilitating reflective occupational performance, understanding of volition, and intentional choice and patterns of doing.

Given that population-based wellbeing requires a holistic approach, collaborations between occupational therapists and behavioral scientists with digital designers, or technology developers should be encouraged. Development of digital environments to support meaningful participation may be an important avenue for development of routines and balance wherein occupations are used as therapeutic means to health and wellbeing.

Digital literacy initiatives should move beyond excessive screen time to how algorithmically curated digital environments influence occupational choices, routines and identity. These may include critical reflection on social comparison, performative culture, and displacement of meaningful occupations while exploring broader societal, regulatory, or technological responses to the underlying issues.

## 6 Conclusion

This perspective proposes an emerging occupational phenomenon in which dopamine-scrolling is more than a pattern of excessive SMU; a factor that reshapes individuals’ occupational choices, meanings, and identities. According to the MOHO, dopamine-scrolling and SMU may influence occupational functioning. Occupational choices may shift towards occupations that reinforce desirable digital identities. This could disrupt or displace occupations and impair occupational balance. These conceptual propositions are interpretive in nature, require further exploration, and evidence-based validation.

## Data Availability

The original contributions presented in the study are included in the article/supplementary material, further inquiries can be directed to the corresponding author.
